# Phonological abilities in literacy-impaired children: Brain potentials reveal deficient phoneme discrimination, but intact prosodic processing

**DOI:** 10.1016/j.dcn.2016.11.007

**Published:** 2016-11-27

**Authors:** Claudia Männel, Gesa Schaadt, Franziska K. Illner, Elke van der Meer, Angela D. Friederici

**Affiliations:** a Max Planck Institute for Human Cognitive and Brain Sciences, Leipzig, Germany; b Department of Psychology, Humboldt-Universität zu Berlin, Germany; c Berlin School of Mind and Brain, Humboldt-Universität zu Berlin, Germany

**Keywords:** Literacy, Phonology, Prosody, Mismatch negativity (MMN), Closure positive shift (CPS)

## Abstract

Intact phonological processing is crucial for successful literacy acquisition. While individuals with difficulties in reading and spelling (i.e., developmental dyslexia) are known to experience deficient phoneme discrimination (i.e., segmental phonology), findings concerning their prosodic processing (i.e., suprasegmental phonology) are controversial. Because there are no behavior-independent studies on the underlying neural correlates of prosodic processing in dyslexia, these controversial findings might be explained by different task demands. To provide an objective behavior-independent picture of segmental and suprasegmental phonological processing in impaired literacy acquisition, we investigated event-related brain potentials during passive listening in typically and poor-spelling German school children. For segmental phonology, we analyzed the Mismatch Negativity (MMN) during vowel length discrimination, capturing automatic auditory deviancy detection in repetitive contexts. For suprasegmental phonology, we analyzed the Closure Positive Shift (CPS) that automatically occurs in response to prosodic boundaries. Our results revealed spelling group differences for the MMN, but not for the CPS, indicating deficient segmental, but intact suprasegmental phonological processing in poor spellers. The present findings point towards a differential role of segmental and suprasegmental phonology in literacy disorders and call for interventions that invigorate impaired literacy by utilizing intact prosody in addition to training deficient phonemic awareness.

## 1 Introduction

Successful literacy crucially depends on intact phonological processing. In learning to read and write children need to be aware of the sounds of their native language, created from *segmental* phonemes as the smallest sound elements, as well as the prosody of *suprasegmental* words and phrases (Bryant and Goswami, 1987). At the segmental level, for example, listeners need to carefully discriminate phonemic details of vowels and consonants to fathom the intended meaning of utterances (e.g., *wet cat* versus *vet cat*). In spelling, the written coding of phonemes follows language-specific rules and is more challenging in languages with less consistent phoneme-grapheme correspondences (Ziegler et al., 2010, Ziegler and Goswami, 2006). In the reverse grapheme-to-phoneme mapping, that is reading, phonemic awareness is particularly relevant for beginning readers, who decipher words sound by sound (Coltheart et al., 2001, Ziegler et al., 2000). Taken together, the ability to successfully detect and manipulate phonemes (i.e., phonemic awareness) is essential in both spelling and reading acquisition and has been reported as predictor of literacy outcome across languages (Caravolas et al., 2013, Moll et al., 2014, Seymour et al., 2003, Ziegler et al., 2010).

Phonemic awareness has not only been found to explain individual differences in reading and spelling, but also to differentiate typical and literacy-impaired populations, as in developmental dyslexia (DD) (World Health Organization, 2009). The idea of intact phonological processing, specifically phonemic awareness, as essential precursor of successful literacy acquisition is rooted in the *phonological deficit theory* of DD (Ramus et al., 2003, Snowling, 2000). While already newborns are able to discriminate different phonemes (Cheour-Luhtanen et al., 1995, Kushnerenko et al., 2001), infants at familial risk of developing DD were found to show impaired discrimination of vowel and consonant lengths (Leppänen et al., 2002, Pihko et al., 1999). Moreover, longitudinal studies have attested a direct relation between infants’ phonemic abilities and later literacy skills (Schaadt et al., 2015, van Zuijen et al., 2013). Also, school children and adults with DD were found to perform significantly worse on phoneme discrimination tasks than their unaffected peers (Groth et al., 2011, Hämäläinen et al., 2009, Landerl, 2003, Steinbrink et al., 2014). Thus, there is a substantial line of research on the, potentially causal, relationship between the perception of segmental phonology and literacy impairments (see also, Goswami, 2015).

Studies on the described phonology-DD relationship carried out in German specifically focused on the relation between vowel length discrimination and spelling (Groth et al., 2011, Landerl, 2003, Steinbrink et al., 2014). This is motivated by the following characteristics of the German language: Duration is an important phonemic feature, because vowel length can subserve lexical distinctions (e.g., *Lamm*/lam/, [lamb] versus *lahm/*la:m/, [lame]). However, there is a high variability in the orthographic representation of vowel lengthening (e.g., *Bahn* [train], *Boot* [boot], or *Lied* [song]). This inconsistent phoneme-to-grapheme correspondence turns correct spelling into a major challenge, even for older children (Klicpera et al., 1993, Klicpera and Gasteiger-Klicpera, 2000). Accordingly, spelling has been found to be more informative than reading (as in German reading, grapheme-to-phoneme correspondences are highly regular), in dissociating German children with DD from their unimpaired peers (Neuhoff et al., 2012, Schaadt et al., 2015, Schaadt et al., 2016). Furthermore, German-speaking adults with DD have often compensated for their initial reading impairments, but typically still encounter severe spelling problems (Cantiani et al., 2013, Landerl and Wimmer, 2008, Männel et al., 2015, Ziegler and Goswami, 2006).

In contrast to segmental phonology dealing with phonemes, suprasegmental phonology or prosody operates at linguistic units spanning several phonemes. Specifically, prosody involves the adequate word- and phrase-level application of stress, timing, and intonation, substantially facilitating listeners’ comprehension. For example, stress placement in words can serve lexical and syntactic distinctions of nouns and verbs (e.g., *object* versus *object*) and support word segmentation from sentence context (Cutler and Norris, 1988). Moreover, the intonation characteristics of sentences signal syntactic units (Nespor and Vogel, 1986, Selkirk, 1984), and prosodic boundaries can aid the resolution of syntactic structure ambiguities (Price et al., 1991, Speer et al., 2011; see also Bögels et al., 2011). The relationship between children’s prosodic skills and reading development has recently received increasing attention (David et al., 2007, Gutierrez-Palma et al., 2009, Holliman et al., 2008, Holliman et al., 2010a, Holliman et al., 2010b, Whalley and Hansen, 2006). For example, prosodic skills were found to predict reading accuracy in English-speaking children, independently of phonemic awareness (Lochrin et al., 2015). In a longitudinal approach, lexical stress sensitivity accounted for children’s reading performance 6–12 months later (Calet et al., 2015, Holliman et al., 2010b). There is also evidence for the role of prosodic skills in spelling, because lexical stress sensitivity predicted children’s spelling scores, independently of phonemic awareness and vocabulary (Wood, 2006). While most of these literacy studies examined prosodic patterns at the lexical level, there is first evidence that different suprasegmental aspects, also at phrase and sentence levels, all make their contribution to reading development (Holliman et al., 2014).

In addition to the well-established role of segmental phonology in DD, suprasegmental phonology is increasingly considered as explaining factor in literacy impairments. For example, Anastasiou and Protopapas (2015) compared segmental and suprasegmental phonological skills in Greek-speaking adolescents with DD and reported impaired segmental awareness and word-level stress assignment in both reading and spelling. In contrast, English-speaking children with DD were not found to differ in word-level stress pattern discrimination from their unaffected peers (Anderson et al., 2013). Most of the studies on suprasegmental phonology focused on the word level, examining lexical stress perception, but there are also investigations of phrasal prosody. Holliman et al. (2012) observed that children with reading difficulties performed below age-matched controls in lexical stress perception, but comparably well in phrasal stress perception. Furthermore, Geiser and colleagues tested intonation processing of children with and without DD and found that both groups benefitted from prosodic phrase boundaries in syntactic ambiguity resolution (Geiser et al., 2014). Together these studies deliver equivocal evidence of prosodic processing in impaired literacy, either reporting deficient prosodic skills (Barry et al., 2012, Goswami et al., 2010, Goswami et al., 2013, Goswami et al., 2002, Leong et al., 2011) or intact prosodic skills (Anderson et al., 2013, Marshall et al., 2009, Mundy and Carroll, 2013, Wood and Terrell, 1998).

Given the controversial findings on the role of prosody in literacy impairments, the current study aims to compare the processing of suprasegmental and segmental phonology in typically spelling and poor-spelling German school children, classified by their writing scores in sentence dictation. We will use the method of event-related brain potentials (ERPs) in two established experimental paradigms, with the advantage of obtaining children’s behavior-independent neural responses underlying different aspects of phonological processing (while additionally controlling for their non-verbal intelligence). First, we will study segmental phonological processing by testing vowel length discrimination, evaluating Mismatch Negativity (MMN) responses for automatic auditory deviancy detection in repetitive contexts (Näätänen et al., 1978). School children with DD have been reported to show reduced MMN amplitudes compared to normally developing peers in response to phoneme changes (Paul et al., 2006), phoneme duration (Lovio et al., 2010), nonverbal sound changes (Lachmann et al., 2005), and duration changes of nonverbal sounds (Corbera et al., 2006), indicating deficient auditory processing (for a review, see Bishop, 2007). In addition to the MMN, some studies on the discrimination of complex auditory stimuli (e.g., syllables) also reported a Late Discriminative Negativity (LDN) (Alonso-Bua et al., 2006, Ceponiene et al., 2002), with an ambiguous picture of LDN modulations in DD: Studies either found divergent LDN amplitudes in affected children compared to their peers (Alonso-Bua et al., 2006, Hämäläinen et al., 2015, Hommet et al., 2009, Neuhoff et al., 2012), or no LDN modulations by DD (Widmann et al., 2012). Because ample evidence revealed attenuated MMN responses to phoneme contrasts in DD, but no consistent LDN findings, we primarily expected the MMN to vowel length variations in our study to be modulated by children’s spelling abilities.

Second, we will study suprasegmental phonological processing by testing prosodic boundary (PB) perception, evaluating the ERP component Closure Positive Shift (CPS). Across languages, the CPS has been reported to occur in response to sentence-level PBs (Steinhauer et al., 1999; for a review, see Bögels et al., 2011) and observed in children from age 3 (Männel and Friederici, 2011). The CPS has been interpreted as reflecting the structuring of auditory input, based on a combination of acoustic modulations, such as changes in pitch and duration, defining prosodic phrases (see Bögels et al., 2011). In contrast to MMN studies, there are no CPS studies in populations with DD yet, such that our study will reveal by means of a behavior-independent electrophysiological measure, whether phrasal prosody perception is deficient or intact in German literacy-impaired children. The former would imply a general phonological impairment, applying to both segmental and suprasegmental levels, while the latter would suggest differential roles of segmental and suprasegmental phonology in DD. In sum, the combined findings of the current ERP studies will substantially contribute to our understanding of different aspects of phonological processing in literacy impairments.

## 2 Materials and methods

### 2.1 Participants

All children were participants of the longitudinal German Language Developmental Study (GLAD-study), starting at children’s birth, investigating the progression of their receptive and expressive language skills. From the original sample, 46 children underwent the standardized German spelling test (***DE****utscher **RE**chtschreib **T**est*; *DERET*; Stock and Schneider, 2008) at 10 years (*M* = 9.83, *SD* = 0.52). In individual test sessions, children were asked to write down ten dictated sentences, applying German phoneme-to-grapheme conversion rules. Sentences were dictated one after another, such that there was no time limit for each sentence to be written down. Spelling errors (i.e., number of words with at least one spelling error) were translated into age-normed percentile ranks (PRs). Children were classified according to the *DERET* norms that define average/above-average performance by a PR ≥ 26 and below-average performance by a PR ≤ 25. Due to the possibility of participants of different groups to “overlap” or to be very close in their spelling performance, we excluded the lowest performing typical speller (PR = 32) and the highest performing poor speller (PR = 25), resulting in a minimum spelling group difference of 11 points on the PR scale. Thus, our participant sample included 22 typical spellers (PR range = 35–93) and 22 poor spellers (PR range 0–24) (for mean/SD values, see Table 1). For the purpose of the present study, both groups were invited at the age of 11 years to participate in the ERP experiments on phonological processing. In addition, to ensure that children’s performance differences are not caused by any differences in general intelligence, we examined children’s non-verbal intelligence by the German version of the ***K****aufmann-**A**ssessment **B**attery for **C**hildren* (*K-ABC*; Kaufman et al., 2009). Performance in five subtests (e.g., *Spatial working memory* and *Matrices*) was translated into age-normed standard scores (*M* = 100, *SD* = 15). As can be seen from Table 1, there were no differences between spelling groups in test age and non-verbal intelligence, while the higher proportion of male than female participants in the poor spellers group reflects the typical sex prevalence of literacy disorders.

**Table 1:** Demographic and literacy variables of typically and poor-spelling children.

	Typical spellers (mean/SD)	Poor spellers (mean/SD)	*t*-values
Demographic variables			
N (female)	22 (10)	22 (6)	
Age (years)	11.06 (0.60)	10.93 (0.70)	n.s.
Non-verbal IQ (SSc)	113.23 (5.96)	110.86 (5.95)	n.s.
			
Spelling^a^			
Spelling from dictation (PR)	65.73 (19.81)	11.14 (7.27)	12.11**

Phonemic awareness^a^			
Phonemic awareness test battery (PR)	62.95 (26.63)	28.18 (23.82)	4.57**

Reading^b^			
Text reading speed (PR)	46.62 (25.23)	24.11 (11.74)	3.47**
Text reading comprehension (PR)	51.24 (27.68)	33.78 (21.11)	2.19*

SSc = Standard scores (*M* = 100, *SD* = 15); PR = percentile rank.

a at 9.83 years (*SD* = 0.53).

b at 12.85 years (*SD* = 0.58) for 21 typical and 18 poor spellers.

* p ≤ 0.05.

** p ≤ 0.001.

All participants were German monolinguals. None of the children had any known hearing deficits or neurological problems. The study followed American Psychological Association (APA) standards in accordance with the declaration of Helsinki from 1964 (World Medical Association, 2013) and was approved by the ethics committee of the University Leipzig. Parental written consent was obtained after children and parents had been in detail informed about the procedure and agreed to participation.

### 2.2 Additional behavioral tests

Text reading was assessed with the standardized German Test of reading speed and reading comprehension (***L****ese**Ge**schwindigkeits- und −**V**erständnis**T**est*; *LGVT*; Schneider et al., 2007), requiring participants to silently read a cloze text as far as possible in four minutes, inserting missing words by choosing from three semantically different options. Reading speed (i.e., number of read words) and comprehension performance (i.e., number of semantically correct insertions) were translated into PRs according to grade-specific norms for school children.

Participants also underwent the standardized test for Basic competences for reading and writing skills to assess phonemic awareness (***BA****sis**KO**mpetenzen für Lese- Rechtschreibleistungen 1–4*; *BAKO 1–4;* Stock et al., 2003) with seven subtests on phoneme manipulation (e.g., pseudoword segmentation and phoneme deletion). PRs are calculated according to norms for school children.

### 2.3 MMN experiment

For the MMN experiment on vowel length processing, a passive listening oddball paradigm was conducted. Participants were presented with a frequently occurring standard syllable, which was occasionally replaced by a deviant syllable. A short-vowel syllable/ba/(202 ms) and a long-vowel syllable/ba:/(431 ms) were used as stimuli. The short-vowel syllable/ba/was recorded in a sound-proof chamber from a female German native speaker and digitized (44.1 kHz/16-bit sampling rate, mono). The steady-state part of the vowel was digitally lengthened to create the long syllable/ba:/(PRAAT; Boersma, 2001; for more detail on the procedure, see Friederici et al., 2002, Friedrich et al., 2004). The actual experiment on vowel length discrimination consisted of two experimental blocks. In one block the short syllable/ba/served as the standard stimulus and the long syllable/ba:/as a deviant stimulus; and vice versa in the other block. The order of blocks was counterbalanced across participants. Within one block, 525 syllables were presented, with 420 standard syllables (83%) and 105 deviant syllables (17%). The inter-stimulus interval (offset to onset) was 855 ms. Stimuli were presented via loudspeaker; and the experiment duration was 20 min.

### 2.4 CPS experiment

**Fig. 1:**
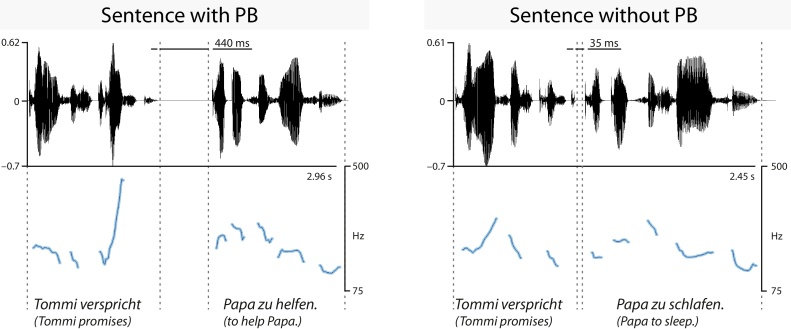
CPS paradigm on prosodic boundary perception. Waveform (normalized values) and pitch track (F0 contour in Hz) of example sentences with and without prosodic boundary (adapted from Männel and Friederici, 2011).

The CPS experiment on PB processing comprised 50 sentences with PBs and 50 sentences without PBs (Fig. 1; for more detail, see Männel and Friederici, 2009, Männel and Friederici, 2011). All sentences were recorded in a sound-proof chamber from a female German native speaker using child-directed speech, digitized (44.1 kHz/16-bit sampling rate, mono) and normalized in amplitude to 70%.

Sentences with and without PBs were constructed in pairs of identical wording up to the sentence-final verb, but differed in their syntactic structure (Fig. 1). The identical word order induces a structural ambiguity (i.e., early or late closure) that is only resolved by the appearance of the last verb. However, listeners can determine the correct sentence structure prior to this point. Intonation and duration characteristics signal the different syntactic units from sentence onset, resulting in sentences with PBs (early closure) and without PBs (late closure). Hence, the current sentence manipulation specifically targets children’s prosodic and syntactic processing, while disregarding lexico-semantic processing (e.g., sentence-related verb processing), which is irrelevant for the sentence parts before and after the PB.

As exemplified in Fig. 1, PBs were realized by distinct acoustic parameters, signaling the presence of preboundary lengthening, boundary-related pitch changes and pausing. Specifically, sentences with PBs were significantly longer (2890 ms; *SD* = 136) than sentences without PBs (2200 ms, *SD* = 145; *t*(98) = −24.71, *p* ≤ 0.001). This duration difference was reflected in the longer first sentence part (i.e., sentence onset to boundary pause onset) of sentences with PBs than without PBs (1078 ms, *SD* = 109 versus 838 ms, *SD* = 114; *t*(98) = −10.78, *p* ≤ 0.001), driven by the longer preboundary syllable in sentences with PBs (*t*(98) = −22.99, *p* ≤ 0.001). Moreover, the preboundary pitch rise was larger (237.00 Hz, *SD* = 49.81 versus 32.42 Hz, *SD* = 21.84; *t*(98) = −36.22, *p* ≤ 0.001) and the pause following the first sentence part was significantly longer (560 ms, *SD* = 26 versus 45 ms, *SD* = 22; *t*(98) = −100.38, *p* ≤ 0.001) for sentences with PBs than without PBs.

Sentences were delivered via loudspeaker and presented in a pseudo-random order (i.e., ≤three succeeding sentences of one prosodic type). Each of the 100 sentences was followed by an inter-stimulus interval of 1.5 s, with an overall experiment duration of 8 min.

### 2.5 EEG recordings and analysis

During the CPS and MMN experiments children sat in an electrically shielded and sound-attenuated booth, while an EEG was recorded. To keep children engaged in listening to the experimental stimuli and prevent them from moving, they watched a silent children’s video (for a similar procedure in preschool and school children, see Männel et al., 2013, Schaadt et al., 2015). The order of the MMN and the CPS experiment was counterbalanced across participants.

**Fig. 2:**
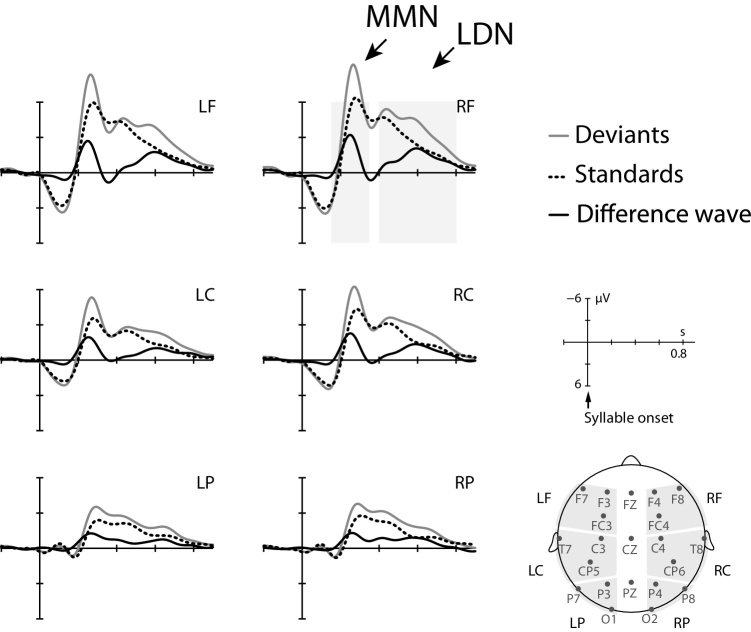
MMN paradigm on vowel length discrimination. ERP responses to standard and deviant stimuli, and ERP difference waves (deviant–standard) relative to syllable onset for both vowel length contrasts (long vowel deviant/ba:/– short vowel standard/ba/and short vowel deviant/ba/– long vowel standard/ba:/) across typically spelling and poor-spelling children. Grey-shaded areas indicate time windows of significant condition effects. As apparent from the difference wave, children show an MMN, followed by an LDN to the vowel length contrasts.

The EEG was recorded with QRefa Acquisition Software, Version 1.0 beta (MPI-CBS, Leipzig, Germany) from 23 Ag/AgCl electrodes attached to elastic caps (Easy Cap GmbH, Germany) at standard positions (see Fig. 2). The electrode montage was identical across experiments and motivated by our previous ERP studies with infants and children (Männel and Friederici, 2009, Männel and Friederici, 2011, Männel and Friederici, 2013, Schaadt et al., 2015, Schaadt et al., 2016). This electrode montage ensured sufficient scalp coverage, while still allowing for short set-up times suitable for experiments with children. Electrooculograms were recorded from electrodes at the outer canthi of both eyes and the orbital ridges of the right eye. EEG recordings were online referenced to CZ; with a ground electrode at FP1. Electrode impedances were mostly kept below 5 kΩ (at least below 10 kΩ). The EEG signal was amplified with the Refa system (Twente Medical Systems International B.V.), digitized online at 500 Hz (anti-aliasing lowpass filter of 135 Hz). Offline, the EEG was algebraically re-referenced from CZ to the average of both mastoids.

For the EEG data analysis of the MMN experiment, a band-pass filter at 0.5–20 Hz (−3dB–cutoff frequencies of 0.59 Hz and 19.91 Hz) and for the CPS experiment, a band-pass filter at 0.2–20 Hz (−3dB–cutoff frequencies of 0.25 Hz and 19.91 Hz) was applied. The use of different filters was motivated by the different ERP components under investigation, such that for the slower CPS component the high-pass filter was less restrictive (at 0.2 Hz) than for the faster MMN (at 0.5 Hz) (see Männel and Friederici, 2009, Männel and Friederici, 2011, Männel and Friederici, 2013, Schaadt et al., 2015, Schaadt et al., 2016). For the MMN experiment, we obtained EEG epochs (so-called trials) of 900 ms post-syllable onset with a pre-stimulus baseline of 200 ms (see also Friederici et al., 2002, Friedrich et al., 2004), and, for the CPS experiment, we obtained trials of 3000 ms relative to sentence onsets with a pre-stimulus baseline of 200 ms (see also Männel and Friederici, 2009, Männel and Friederici, 2011, Männel et al., 2013). For both experiments, prototypical blinks and eye-movements were identified for each participant and used as an individual correction template for trials containing blink and eye-movement artifacts (correlation-based correction algorithm implemented in EEP 3.3, MPI-CBS, Germany). Regarding all other artifacts, trials with a standard deviation exceeding 80 μV within a 200 ms-sliding window were automatically rejected. Moreover, for the MMN experiment, standard trials presented directly after a deviant trial were excluded from further analyses. Poor-spelling and typically spelling children did not differ significantly concerning the number of artifact-free MMN deviant trials neither for the short syllable/ba/ (87.26%, *SD* = 11.56 vs. 84.11%, *SD* = 10.11), nor for the long syllable/ba:/ (88.44%, *SD* = 13.53 vs. 84.11%, *SD* = 11.11). Spelling groups did also not differ regarding the number of trials for sentences with PB (71.91%, *SD* = 16.98 vs. 70.54%, *SD* = 16.55), nor for sentences without PB (72.91%, *SD* = 14.87 vs. 67.18%, *SD* = 17.33).

### 2.6 Statistical analyses

**Fig. 3:**
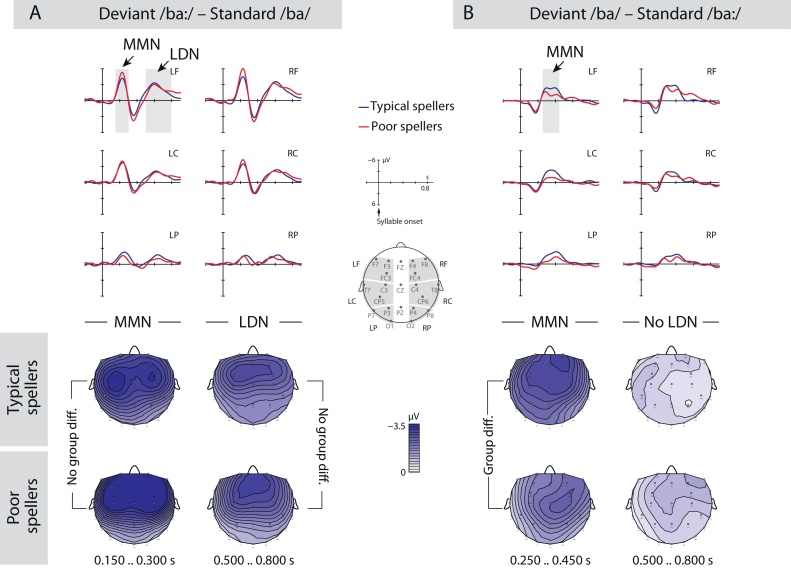
MMN paradigm on vowel length discrimination. ERP difference waves and difference maps (deviant–standard) of typically spelling and poor-spelling children relative to syllable onset for A) the contrast long vowel deviant/ba:/– short vowel standard/ba/and B) the contrast short vowel deviant/ba/– long vowel standard/ba:/. Grey-shaded areas indicate time windows of significant condition effects. Both spelling groups show an MMN to both vowel length contrasts, but an LDN for the contrast long vowel deviant/ba:/– short vowel standard/ba/only. The MMN of poor spellers is less pronounced for the contrast deviant/ba/- standard/ba:/compared to the MMN of typical spellers. No differences between spelling groups were found for the contrast deviant/ba:/- standard/ba/.

**Fig. 4:**
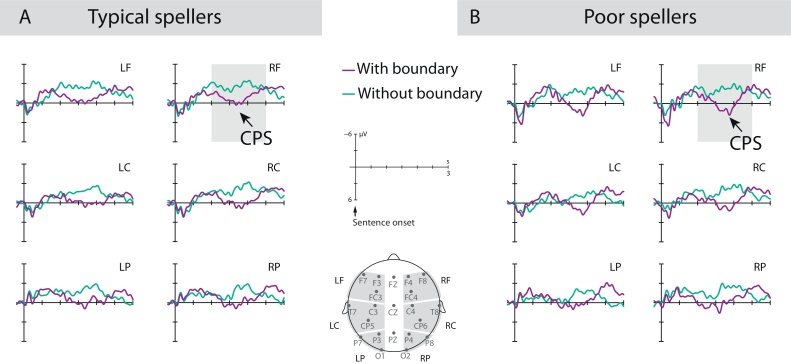
CPS paradigm on prosodic boundary perception. ERPs of A) Typical spellers and B) Poor spellers relative to sentence onset for sentences with and without prosodic boundaries (PBs). Grey-shaded areas indicate time windows of significant sentence type effects. Both spelling groups show CPS in responses to PBs without any statistical differences.

Statistical analyses were separately performed for mean amplitudes recorded at midline and lateral electrodes across defined time windows (TWs). For lateral sites, six regions of interest (ROIs) were created combining hemisphere (left, right) and region information (anterior, central, posterior) (see Fig. 2, Fig. 3, Fig. 4).

For the MMN experiment, visual inspection of the average differences wave (deviant–standard) across both spelling groups, revealed two ERP effects of different latency ranges: an early negative peak and a later longer-extended negative deflection. TWs for statistical analyses were defined relative to the peaks of both components. In line with the ERP findings of previous auditory oddball studies, the early peaking component (150–350 ms) is referred to as MMN and the later deflection (400–800 ms) as LDN (see Neuhoff et al., 2012, Schulte-Körne et al., 1998, Schulte-Körne et al., 2001). An initial omnibus analysis of variance (ANOVA) with the within-subject factors phoneme (long vowel deviant, short vowel deviant), condition (deviant, standard), region, and hemisphere and the between-subject factor spelling group (typical spellers, poor spellers) was calculated to determine whether there were condition differences depending on the phoneme contrast (i.e., experimental runs of long vowel deviants among short standards versus short vowel deviants among long standards) and if so, subsequent analyses were calculated separately for each contrast, defined as follows: For midline electrodes, three-way ANOVAs included the factors condition, electrode site (Fz, Cz, Pz) and spelling group, while for lateral ROIs, four-way ANOVAs included the factors condition, region, hemisphere, and spelling group. All significant effects (Greenhouse-Geisser-corrected) involving the factor condition are reported; and significant interactions with the factor condition were further analyzed using one-sample *t*-tests (Bonferroni-corrected).

For the CPS experiment, mean ERP amplitudes were analyzed covering the average sentence length with the following post-sentence onset TWs: TW 0–500, TW 500–1000, TW 1000–1500, TW 1500–2000, TW 2000–2500, and TW 2500–3000 (see Männel and Friederici, 2009, Männel and Friederici, 2011, Männel et al., 2013). At midline electrodes, three-way ANOVAs with the factors sentence type (with PBs, without PBs), electrode site, and spelling group were run on each TW. For the lateral ROIs, four-way ANOVAs with the factors sentence type, region, hemisphere, and spelling group were run on each TW. All significant effects (Greenhouse-Geisser-corrected) involving the factor sentence type are reported; and significant interactions with sentence type were further analyzed using one-sample *t*-tests (Bonferroni-corrected).

## 3 Results

### 3.1 Behavioral tests

As documented in Table 1, typical spellers and poor spellers did not only per definition significantly differ in their spelling performance, but also in additional literacy tasks, namely phonemic awareness and text reading (i.e., reading speed and comprehension). In general, poor spellers showed significantly lower phonemic awareness abilities, reading accuracy, and slower reading speed compared to typical spellers.

### 3.2 MMN experiment

As apparent from Fig. 2, difference waves of ERP responses to deviant syllables minus standard syllables across both phoneme contrasts (i.e., different experimental runs of short vowel deviants among long standards versus long vowel deviants among short standards) revealed two negative deflections, an early negativity in the MMN time range and a late negativity in the LDN time range. Accordingly, TWs for statistical analyses were defined with respect to the average MMN peak (+/−100 ms) at 150–350 ms and the average LDN peak (+/−200 ms) at 400–800 ms. The omnibus ANOVA with the within-subject factors phoneme, condition, region, and hemisphere and the between-subject factor spelling group (typical spellers, poor spellers) revealed for both TWs condition differences depending on the phoneme contrast (TW 150–300 ms: F(1,42) = 20.05, *p* < 0.001; TW 400–800 ms: F(1,42) = 13.92, *p* < 0.001), indicating differences in the modulation of MMN and LDN depending on the experimental run. Subsequent analyses were thus separately calculated for each phoneme contrast, and TWs were adjusted accordingly (long vowel deviant: MMN 150–300 ms, LDN 500–800 ms; short vowel deviant: MMN 250–450 ms, LDN 500–800 ms).

The ANOVA for the long vowel deviant confirmed the presence of an MMN (TW 150–300 ms) with a fronto-central distribution at lateral ROIs and midline sites, but no LDN (TW 500–800 ms) (Fig. 3A, Table 2 for statistically significant effects). Notably, none of these condition effects involved interactions with the factor spelling group, indicating comparable discrimination of long vowel-syllables among short vowel-syllables in typical and poor spellers.

**Table 2:** MMN experiment with long vowel deviant: ANOVAs for mean amplitudes in MMN and LDN time windows relative to syllable onset.

TWs (ms)	Lateral ROIs			Midline sites		
	*Effect*	*df*	*F/t*	*Effect*	*df*	*F/t*
MMN: 150–300	Cond	1,42	96.17**	Cond	1,42	81.54**
	Cond x Reg Fron Cen Pos	2,84 1,43 1,43 1,43	28.87** −8.29** −9.98** −6.49**	Cond x Site FZ CZ PZ	2,84 1,43 1,43 1,43	16.63** −8.40** −8.96** −7.59**
	Cond x Reg x Hem Left Fron Left Cen Left Pos Right Fron Right Cen Right Pos	2,84 1,43 1,43 1,43 1,43 1,43 1,43	5.93* −7.73** −9.66** −6.15** −8.22** −9.02** −5.59**	a		
LDN: 500–800	Cond	1,42	81.26**	Cond	1,42	83.05**
	Cond x Reg Fron Cen Pos	2,84 1,43 1,43 1,43	24.42** −9.10** −8.83** −4.84**	Cond x Site FZ CZ PZ	2,84 1,43 1,43 1,43	24.37** −9.16** −9.07** −5.63**

Cond = condition (deviant *vs* standard); Reg = region; Fron = frontal; Cen = central; Pos = posterior; Site = electrode site; Hem = hemisphere.

Note that only statistically significant effects are reported.

a The interaction Cond × Region × Spelling Group *F*(2,88) = 4.72 did not reveal any significant step-down results involving the factor spelling group and was thus disregarded.

* p ≤ 0.01.

** p ≤ 0.001.

The ANOVA for the short vowel deviant also attested the presence of broadly distributed MMN (TW 250–450 ms) and LDN (TW 500–800 ms), however with less pronounced amplitudes than in the long vowel condition (Fig. 3B, Table 3 for statistically significant effects). Importantly, for the earlier TW, there was a condition x spelling group interaction, with the step-down analyses demonstrating a condition effect in both groups that, however, was less pronounced in poor-spelling children (two-sample *t* test; *t*(42) = 2.23, *p* < 0.05). Thus, for the discrimination of short vowel-syllables among long vowel-syllables analyses revealed differences between spelling groups, such that poor spellers showed a diminished MMN relative to typical spellers.

**Table 3:** MMN experiment with short vowel deviant: ANOVAs for mean amplitudes in the MMN time window relative to syllable onset.

TWs (ms)	Lateral ROIs			Midline sites		
	Effect	df	F/t	Effect	df	F/t
MMN: 250–450	Cond	1,44	81.97***	Cond	1,44	70.17***
	Cond x Group Typical Spellers Poor spellers	2,84 1,21 1,21	4.95* −8.06*** −4.78***			
	Cond x Reg Fron Cen Pos	2,84 1,43 1,43 1,43	5.25* −6.89*** −7.24*** −6.73***			
	Cond x Reg x Hem Left Fron Left Cen Left Pos Right Fron Right Cen Right Pos	2,84 1,45 1,45 1,45 1,45 1,45 1,45	6.24** −5.35*** −5.86*** −6.28*** −7.82*** −6.64*** −5.60***			

Cond = condition (deviant *vs* standard); Reg = region; Fron = frontal; Cen = central; Pos = posterior; Hem = hemisphere.

Note that only statistically significant effects are reported.

* p ≤ 0.05.

** p ≤ 0.005.

*** p ≤ 0.001.

### 3.3 CPS experiment

For both spelling groups, ERP responses revealed a positive shift (starting at ∼1000 ms post-sentence onset) in response to sentences with PBs in contrast to sentences without PBs (Fig. 4). This observation was statistically confirmed in ANOVAs with post-sentence onset effects of sentence type starting from TW 1000–1500 ms onwards (Table 4 for statistically significant effects). Note, however, that the effect of sentence type in the TW 2500–3000 ms is not considered as result of boundary processing, but driven by the intonation contour signaling the sentence end of the shorter sentences without PBs prior to the sentences with PBs. Importantly, none of the statistical contrasts revealed a significant interaction with spelling group. Thus, statistical analyses evidenced for both typical and poor spellers comparable differences in the processing of the two sentence types, appearing as a broadly distributed positive shift for sentences with PBs.

**Table 4:** CPS experiment: ANOVAs for mean amplitudes across the time range of 0–3000 ms relative to sentence onset.

TWs (ms)	Lateral ROIs			Midline sites		
	Effect	df	F/t	Effect	df	F/t
1000–1500	Sentence	1,42	4.67*	Sentence	1,42	22.36***
				Sentence x Site FZ CZ PZ	2,84 1,43 1,43 1,43	4.17* 4.60*** 4.54*** 3.18**
1500–2000	Sentence	1,42	32.09***	Sentence	1,42	69.07***
				Sentence x Site FZ CZ PZ	2,84 1,43 1,43 1,43	6.92** 7.12*** 8.30*** 6.29***
2000–2500	Sentence	1,42	5.44*			
2500–3000	Sentence	1,42	13.11***	Sentence	1,42	15.11***
				Sentence x Site CZ PZ	2,84 1,43 1,43	4.27* 3.17** 5.40***

Sentence = sentence type.

Note that only statistically significant effects are reported.

* p ≤ 0.05.

** p ≤ 0.005.

*** p ≤ 0.001.

## 4 Discussion

The present ERP study examined the processing of segmental and suprasegmental phonology in poor-spelling and typically spelling German school children. Segmental phonology was studied by evaluating the MMN in vowel length discrimination (Näätänen et al., 1978), while suprasegmental phonology was studied by evaluating the CPS in PB perception (Steinhauer et al., 1999).

Importantly, the MMN and the CPS experiments revealed opposing results, evident in either deficient or intact phonological processing in children with spelling problems compared to their unaffected peers. For the MMN experiment, both typical spellers and poor spellers similarly showed an MMN and an LDN in discriminating long vowel deviants from short vowel standards with typical latencies for children at this age (Ceponiene et al., 2002). For the reverse discrimination of short vowel deviants from long vowel standards, both groups showed an MMN, but poor spellers’ MMN was significantly diminished as compared to typical spellers. In contrast to the MMN experiment, there were no spelling group differences for the CPS experiment. Both typical and poor spellers similarly showed a broadly distributed CPS in response to PBs. Taken together, our results revealed deficient processing of segmental phonology, but intact suprasegmental phonology (i.e., prosody) in poor-spelling children compared to typically spelling children.

### 4.1 Deficient vowel length discrimination (MMN)

According to the *phonological deficit theory* of DD, intact phonemic awareness is an essential precursor of literacy acquisition (Ramus et al., 2003, Snowling, 2000). Individuals with DD have been repeatedly reported to show deficient phoneme perception and discrimination (see Bishop, 2007), investigated by the MMN as an indicator of auditory processing capacities (Näätänen et al., 2007). Moreover, there is longitudinal evidence on the association of infants’ early phonemic abilities and their later spelling and reading skills (Schaadt et al., 2015, van Zuijen et al., 2013).

For the German language, the successful perception of vowel length information is specifically important as it can subserve lexical distinctions. The correct orthographic representation of vowel length, however, allows more than one form and thus, is challenging even for older children (Klicpera et al., 1993, Klicpera and Gasteiger-Klicpera, 2000). Accordingly, German-speaking children with DD were found to show deficient vowel length categorization (Landerl, 2003) and vowel length discrimination (Groth et al., 2011, Steinbrink et al., 2014). The results of our study suggest that these deficits are grounded in deficient auditory processing, as reflected in MMN amplitude modulations. In the current study, we found reduced MMN amplitudes in poor-spelling children in response to vowel length changes compared to typically spelling children. Similarly, a study in Finnish reported reduced MMN amplitudes in response to vowel length changes in 6-year-old preschool children at risk of DD compared to their normally developing peers (Lovio et al., 2010). These neural precursors were already observed in infancy, as 6-month-olds at risk of DD differed in their Mismatch Responses to vowel length changes from 6-month-olds not at risk of DD (Pihko et al., 1999; see also Leppänen et al., 2002). Together these data suggest a causal relationship between vowel length processing in early development and later literacy acquisition, especially for languages, in which vowel length information subserves lexical distinctions (e.g., German and Finnish).

It is worth noting that we only found MMN amplitude differences between poor spellers and typical spellers when analyzing the short deviant vowel (among long vowels), but not when analyzing the long deviant vowel (among short vowels). These results are in line with previous observations in German infants suggesting the discrimination of short deviant vowels to be more difficult than the processing of long deviant vowels (Friedrich et al., 2004). A reason for these findings might be that the short deviant syllable mainly consist of the long syllable’s features, whereas the long syllable contains additional features, making it perceptually more salient, and, thus, easier to discriminate compared to the short syllables. Adding to this, even school children still demonstrate more difficulties in the behavioral categorization of short vowels compared to long vowels (Landerl, 2003). The notion of stimulus-dependent differences in discrimination difficulty is further supported by our observation that across groups, the short deviant vowel generally evoked smaller MMN and no LDN amplitudes and longer MMN latencies than the long deviant vowel. Reduced MMN amplitudes and longer MMN latencies have been associated with more difficult discrimination (Friedrich et al., 2004, Näätänen and Alho, 1995, Näätänen et al., 2007). Reduced LDN amplitudes, however, have been inter alia interpreted as reduced attention directed towards the deviant stimulus (Mueller et al., 2008) or diminished support of long-term memory representations in deviancy detection (Zachau et al., 2005). Taken together, our data suggest that at the age of 11 years, poor-spelling children might have partially compensated for their deficient phoneme discrimination, as apparent under easier discrimination conditions, but still show these deficits under more challenging conditions.

In contrast to the spelling group differences for vowel length discrimination indicated by the MMN, we did not find any group differences for the LDN. Previous studies on auditory discrimination in literacy-impaired populations delivered an equivocal picture with respect to LDN modulations: For example, Widmann and colleagues found no LDN differences between school children with and without DD (Widmann et al., 2012); whereas Neuhoff and colleagues reported significantly reduced LDN amplitudes in children with DD compared to typically developing peers (Neuhoff et al., 2012). One of the factors explaining these diverging findings might be maturation, because Hommet et al. (2009) did not find any significant LDN differences between adults with and without DD, but between 10-year-olds with and without DD.

In sum, the current MMN data of phoneme length discrimination are in line with previous findings reporting deficient phoneme discrimination in individuals with DD (for a comprehensive overview, see Bishop, 2007) and are in line with the assumption that vowel length discrimination abilities play a major role in literacy acquisition, especially in the German language (Groth et al., 2011, Steinbrink et al., 2014).

### 4.2 Intact prosodic boundary perception (CPS)

Over and above segmental phonology, suprasegmental phonology has been shown to contribute to children’s successful literacy acquisition (Calet et al., 2015, David et al., 2007, Gutierrez-Palma et al., 2009, Holliman et al., 2014, Holliman et al., 2008, Holliman et al., 2010a, Holliman et al., 2010b, Lochrin et al., 2015, Whalley and Hansen, 2006, Wood, 2006). However, studies on prosodic processing in literacy-impaired populations have so far delivered an ambiguous picture: Some studies reported deficient prosody perception in individuals with DD (Barry et al., 2012, Goswami et al., 2010, Goswami et al., 2013, Goswami et al., 2002, Leong et al., 2011), whereas others observed intact prosodic processing (Anderson et al., 2013, Marshall et al., 2009, Mundy and Carroll, 2013, Wood and Terrell, 1998). Not simplifying matters, investigations of prosodic processing in DD at the brain level are missing, despite the growing number of behavioral studies. Therefore, we examined the behavior-independent ERP component CPS in response to PBs in the same samples of poor-spelling and typically spelling children, ensuring comparability to our MMN study on segmental phonological processing under passive task conditions.

Notably, in the present CPS study, we did not find any differences between poor and typical spellers concerning the processing of PBs. This is in line with behavioral evidence showing that school children with DD utilize prosodic phrase information for resolving syntactic structure ambiguities in the same manner as typically developing children (Geiser et al., 2014). At first view, these results seem to contradict findings on dyslexic individuals’ problems in prosodic tasks (Barry et al., 2012, Goswami et al., 2010, Goswami et al., 2013, Leong et al., 2011). However, an evaluation of the particular type of prosodic unit under consideration might reconcile current and previous findings. Indeed, individuals with DD frequently experience intact phrase-level prosody, as evident in successful phrase-level stress perception (Holliman et al., 2012) and sentence-level boundary perception (Anastasiou and Protopapas, 2015, Geiser et al., 2014). In contrast, deficits are often reported for word-level stress assignment in individuals with DD (Barry et al., 2012, Goswami et al., 2013, Holliman et al., 2012). The apparent processing advantage of phrase- and sentence-level prosody over word-level prosody might be grounded in the more prominent acoustic marking of larger prosodic units, present in both magnitude and number of acoustic cues. For example, word-level stress in German is mainly realized by vowel duration and only to some extend by increased pitch and intensity values of the prominent syllable (Dogil, 1995). In contrast, intonational phrases and their defining boundaries are prominently marked by the defining intonation contour leading to a boundary-related pitch change, a lengthening of the preboundary syllable and an extended boundary pause (Nespor and Vogel, 1986, Peters et al., 2005). Furthermore, processing of larger prosodic phrases might be facilitated by the additional linguistic information inherent in larger units. This receives support from a study by Barry et al. (2012) showing that adults with DD benefit from sentence contexts in their decision about word-level stress patterns.

In addition to the role of the prosodic unit under investigation, the influence of task demands seems to be an explaining factor of the opposing findings on prosodic skills in DD. Specifically for word-level prosodic processing, individuals with DD have been shown to perform normally in implicit tasks (e.g., priming tasks, Mundy and Carroll, 2012, 2013), but experience problems in explicit tasks (e.g., reiterative speech tasks, Goswami et al., 2010, Leong et al., 2011). Furthermore, deficient processing has been shown to arise in tasks with increasing memory load (Soroli et al., 2010), matching the well-documented finding of working memory deficits in children and adults with DD (Baddeley and Wilson, 1993, Jorm, 1983, Snowling, 1998; see also Männel et al., 2015). Thus, our finding of intact PB processing in children with DD might be rooted in both the processing advantage for larger prosodic units as well as the advantage of obtaining brain responses during passive listening, thus avoiding intervening task demands.

When discussing factors that influence the outcome of prosodic processing in children with different spelling abilities, the close relation between prosodic and syntactic phrase structure needs to be considered. Prosodic boundaries typically coincide with syntactic boundaries, signaling syntactic units (Nespor and Vogel, 1986, Selkirk, 1984) and supporting the resolution of syntactic structure ambiguities (see Bögels et al., 2011). Thus, children’s CPS in response to PBs might not only be driven by children’s prosodic processing abilities, but also influenced by their syntactic skills. Indeed, children with DD were reported to show impaired comprehension and production of complex syntactic structures (Guasti et al., 2015, Leikin and Assayag-Bouskila, 2004), as well as altered ERP indicators for syntactic phrase structure violations (Sabisch et al., 2006). These deficits, however, do not seem to be reflected in our current ERP data on PB processing, since we did not observe CPS differences between poor and typical spellers.

### 4.3 General discussion

Our study is the first to investigate the neural correlates of segmental and suprasegmental phonological processing in the same sample of literacy-impaired children. Across two ERP experiments, we observed that poor-spelling children, compared to typical spellers, experience difficulties in vowel length discrimination, but show intact PB processing. Thus, our findings point to a differential role of segmental and suprasegmental phonology in literacy disorders.

There is ample evidence indicating a differential involvement of left- and right- hemispheric brain regions in the processing of segmental and suprasegmental aspects of language, respectively (see Friederici, 2011, Friederici and Alter, 2004, Lindell, 2006, Poeppel, 2003). Regarding language processing in DD, children and adults were found to show aberrant left-hemispheric functional activation patterns (for a review, see Richlan et al., 2009). Thus, the results of the present study potentially indicate left hemisphere-specific processing problems in literacy-impaired children, as we observed group differences for segmental phonological processing (typically left-lateralized; e.g., Minagawa-Kawai et al., 2007), but not for suprasegmental processing (typically right-lateralized; e.g., Homae et al., 2007).

However, the observed dissociation in poor-spelling children’s phonological processing might also be nourished in the different ways in which segmental and suprasegmental speech units are acoustically coded. Specifically, in our MMN paradigm on segmental processing, we studied phonemes and evaluated the discrimination of vowel length, which is a duration parameter. In the CPS paradigm on suprasegmental processing we studied prosodic phrases that are defined by prosodic boundaries marked by duration as well as pitch cues (Nespor and Vogel, 1986, Peters et al., 2005). Thus, one could argue that segmental processing difficulties in poor-spelling children can be explained first, by the fewer number of acoustic cues signaling phoneme changes than prosodic phrasing, and, second, the different extension of these acoustic cues spanning short or long speech segments. Notably, our finding of deficient vowel length discrimination also adds to previous evidence of dyslexic individuals’ impaired duration information processing (e.g., Chobert et al., 2012, Goswami et al., 2011). However, deficient duration processing alone cannot explain the current results, because differences in segmental processing between individuals with and without DD have also been observed for pitch manipulations (e.g., Halliday and Bishop, 2006, Maurer et al., 2003) and other phonetic features, as in consonant changes (e.g., Lovio et al., 2010). Thus, together with our study, these findings speak for a general deficit in the processing of smaller segmental units, compared to larger suprasegmental units in literacy disorders, possibly grounded in left hemisphere-specific processing problems.^3^

If our results are confirmed in future studies, the observed dissociation between the processing of segmental and suprasegmental phonology in DD might be meaningful for interventions. There is longitudinal evidence showing that both prosodic skills and phonemic awareness predict reading development (Goswami et al., 2013). Moreover, when modelling causal connections between different aspects of literacy acquisition, Holliman et al. (2014) found that prosodic skills significantly contributed via phonological awareness to reading and spelling acquisition. Given our findings that segmental, but not suprasegmental phonology is impaired in poor-spelling children, it could be argued that the application of prosody-based interventions further enhances the already well-developed suprasegmental phonology, and, thus, supports deficient segmental phonology (see Holliman et al., 2014). This approach might be a promising complement to conventional phonological-awareness trainings in impaired literacy acquisition, especially for cases in which the latter do not take effect (Torgesen, 2000). Prosody training could, for example, follow procedures applied in *ProsA*, a computer-based test for the assessment of receptive and expressive prosody skills from age 4, serving diagnostic and intervention purposes (Walther and Otten, 2015; German adaption of the *PEPS-C*; *Profiling Elements of Prosody in Speech-Communication*; Peppé and McCann, 2003). The *ProsA* comprises five subtests that target different aspects of prosody: sentence mode, word boundaries, sentence focus, affective prosody, and auditory discrimination of prosodic features (pitch, pauses, emphasis). A prosody-based intervention in DD seems especially promising, because children employ prosodic skills in language acquisition from early on, such that at 4 months of age, infants already prefer their native-language specific word-stress pattern (Friederici et al., 2007) and at 6–9 months, they use sentence-level prosodic modulations in lexical segmentation (Männel and Friederici, 2010, Männel and Friederici, 2013).

## Footnotes

3 Note, however, that in order to entirely exclude an alternative explanation of particular duration processing problems in DD, future ERP studies should study PB processing by removing the typical pitch information from the boundary (such that only duration information remains) to confirm our finding of no CPS differences between poor-spelling and typically spelling children.
